# Comparison of administration of single- and triple-course steroid pulse therapy combined with tonsillectomy for immunoglobulin A nephropathy

**DOI:** 10.1097/MD.0000000000027778

**Published:** 2021-12-17

**Authors:** Kanako Watanabe-Kusunoki, Daigo Nakazawa, Junya Yamamoto, Naoko Matsuoka, Nobuharu Kaneshima, Tasuku Nakagaki, Rie Yamamoto, Tomochika Maoka, Sari Iwasaki, Takahiro Tsuji, Yuichiro Fukasawa, Naoki Nishimoto, Saori Nishio, Tatsuya Atsumi

**Affiliations:** a Department of Rheumatology, Endocrinology and Nephrology, Faculty of Medicine and Graduate School of Medicine, Hokkaido University, Sapporo, Japan; b Department of Nephrology, NTT Medical Center Sapporo, Sapporo, Japan; c Department of Pathology, Sapporo City General Hospital, Sapporo, Japan; d Division of Biostatistics, Clinical Research and Medical Innovation Center, Hokkaido University Hospital, Sapporo, Japan.

**Keywords:** immunoglobulin A nephropathy, relapse, remission, steroid pulse therapy, tonsillectomy

## Abstract

Immunoglobulin A nephropathy (IgAN) is a form of chronic glomerulonephritis that can cause end-stage renal disease. Recently, tonsillectomy combined with corticosteroid pulse (TSP) has been shown to be effective for achieving clinical remission and favorable renal outcome in patients with IgAN. However, the standard regimen of corticosteroid use in TSP has not been established. Herein, we compared the effect of single- or triple-course steroid pulse therapy combined with tonsillectomy in patients with IgAN.

This retrospective, observational cohort study included 122 patients with IgAN enrolled from January 2004 to December 2018 at 2 independent institutions. We divided the patients into 2 groups; single-course (TSP1: n = 70) and triple-course (TSP3: n = 52) of corticosteroid pulse therapy (1 course comprised 3 consecutive days’ infusion of 0.5 g methylprednisolone) combined with tonsillectomy. The primary outcome for renal survival was defined as the first occurrence of ≧30% decrease in estimated glomerular filtration rate from baseline. Secondary outcomes included the incidence of clinical remission and recurrence of the disease.

Regarding clinical parameters and findings at baseline, there were no significant differences between the 2 groups. The 8-years renal survival in the 2 groups was not significantly different according to Kaplan–Meier curves (TSP1; 82.5% vs TSP3; 69.2%, log-rank test *P* = .39). The cumulative incidence rates of remission of hematuria (94.4% vs 85.4%, *P* = .56) and clinical remission (85.0% vs 64.8%, *P* = .07) were comparable in both groups, while those of proteinuria showed higher rates in TSP1 than TSP3 (88.4% vs 65.4%, *P* = .02). The cumulative incidence of relapse of hematuria (5.6% vs 2.3%, *P* = .42) and proteinuria (7.1% vs 3.3%, *P* = .41) showed no significant differences in the 2 groups. Cox regression analyses showed that the number of courses of corticosteroid pulse therapy was not significantly associated with renal outcome (TSP1 vs TSP3; Hazard ratios 0.69, 95% confidence intervals 0.29-1.64, *P* = .39).

The effect of single-course corticosteroid pulse therapy is not statistically, significantly different from triple-course in TSP protocol for improving renal outcome and preventing relapse in patients with IgAN. Single-course corticosteroid pulse therapy may become a treatment option for patients with IgAN.

## 1 Introduction

Immunoglobulin A nephropathy (IgAN) is the most common form of primary chronic glomerulonephritis characterized by IgA glomerular deposition and mesangial proliferation. Most patients present with slow progression of the disease with microscopic hematuria and proteinuria. However, 20% to 40% of patients progress to end-stage renal disease within 20 years.^[1]^ IgAN is a complex multifactorial disease, which might be associated with continuous mucosal infection and impaired immune regulation, resulting in glomerular injury with the deposition of IgA1 immune complexes.^[2]^ Based on these assumptions, over the last few decades, various therapeutic interventions have been attempted on patients with IgAN.^[3]^ According to the Kidney Disease Improving Global Outcomes guidelines, the supportive care including blood pressure management with renin-angiotensin system inhibitor (RAS-i) and lifestyle modification is recommended for patients with IgAN as a first line. While, the immunosuppressive therapy is considered for patients with IgAN who are at high risk of progressive chronic kidney disease (CKD) despite maximal supportive care. Corticosteroids are key in the management of IgAN for reducing proteinuria and preventing end-stage renal disease.^[4]^ Tonsillectomy is also reportedly effective for patients with IgAN.^[5,6]^ A randomized controlled study showed that tonsillectomy exhibited considerable improvement in the time to reach the first remission, cumulative remission rate, duration of the first remission, and relapse rate for both hematuria and proteinuria, compared to the control group with supportive therapy alone.^[7]^ A recent large cohort study reported that tonsillectomy improved renal survival independent of the use of RAS-i and corticosteroid.^[8]^ Furthermore, tonsillectomy combined with corticosteroid therapy has additional effects toward achieving clinical remission.^[9,10]^ However, the ideal dosage of corticosteroid, when combined with tonsillectomy, remains controversial. According to the protocol described by Hotta et al,^[9]^ patients receive triple-course intravenous methylprednisolone (0.5 g/d for 3 days), followed by 0.6 mg/kg of oral prednisolone every other day with sequential tapering over 1 year. Because high-dose corticosteroid therapy leads to serious adverse effects including infection, osteoporosis and impaired glucose metabolism,^[11,12]^ the corticosteroid should be administered in anticipation of the minimum requirement.

We aimed to clarify whether the number of corticosteroid pulses affects the therapeutic outcome in patients with IgAN.

## 2 Methods

### 2.1 Patients

We retrospectively enrolled all IgAN patients who were treated with a combination of tonsillectomy and either single (TSP1) or triple (TSP3) courses corticosteroid pulse therapy, from January 2004 to December 2018 at Hokkaido University Hospital and NTT Medical Center Sapporo. Patients were 16 years old or older and had biopsy-proven IgAN. The pathological diagnosis was made by at least 2 pathologists at Sapporo City General Hospital, based on the light microscopic findings and the immunofluorescence analysis of mesangial IgA deposition. Exclusion criteria comprised as follows: observation period <2 years, a previous immunosuppressant therapy including corticosteroids and other immunosuppressive agents, serum creatinine (sCr) at baseline of >5.0 mg/dL, the presence of complications including other renal diseases, previous history of renal transplantation and alternative corticosteroid regimens. Patients with proteinuria, hematuria, and active histological findings were considered to be an active state of IgAN, and received TSP based on informed consent. The number of courses of corticosteroid pulse therapy was determined by each physician's perspective, then the patients were assigned to receive TSP1 or TSP3 treatment. This retrospective observational cohort study was conducted in accordance with the Declaration of Helsinki and approved by the Medical Ethics Committee of Hokkaido University Hospital (019-0085) and NTT Medical Center Sapporo (19-00676).

### 2.2 Treatment protocol

Patients underwent single-course (TSP1) or triple-course (TSP3) intravenous methylprednisolone pulse therapy of 0.5 g/d for 3 consecutive days within 6 months from the beginning of treatment, followed by oral prednisolone (0.6 mg/kg administered every other day). Oral prednisolone was tapered down by 5 mg every 8 weeks and discontinued within 1 year, by reference to the treatment regimen described by Hotta et al^[9]^ In TSP3 group, the second and third timing of corticosteroid pulse was scheduled by the physician's clinical decision within 6 months, including 3 consecutive days for 3 consecutive weeks or those bimonthly for 3 times. Tonsillectomy was performed before or within 1 year after corticosteroid administration.

### 2.3 Evaluation of clinical and laboratory data

Laboratory data and clinical findings at the beginning of corticosteroid pulse administration were recorded as baseline. We included as follows: gender, age, body mass index, systolic and diastolic blood pressure, proteinuria, hematuria, sCr, estimated glomerular filtration rate (eGFR), stage of chronic kidney disease, clinical grade based on criteria of the Japanese Society of Nephrology (grade 1: proteinuria <0.5 g/d, grade 2: eGFR ≧60 mL/min/1.73 m^2^ and proteinuria ≧0.5 g/d, grade 3: eGFR <60 mL/min and proteinuria ≧0.5 g/d), serum albumin (Alb), serum IgA, serum complement 3 (C3), IgA/C3 ratio, hemoglobin A1c, and administration of renin RAS-i at baseline. The number of urinary sediment red blood cells was scored as follows: <1/ high power field (HPF); 1, 1-4/HPF; 2, 5-9/HPF; 3, 10-19/HPF; 4, 20-29/HPF; 5, 30-49/HPF; 6, 50-99/HPF; 7, ≧100/HPF; 8. eGFR was computed according to the equation previously reported.^[13]^ The following equation (modified from the Modification of Diet in Renal Disease equation) was used to estimate eGFR based on serum creatinine value: eGFR (mL/min/1.73 m^2^) = 194 × (serum creatinine [mg/dL]) − 1.094 × (age[years]) − 0.287 (× 0.739 if female). Histological grading was performed according to the Oxford classification^[14]^ and Clinical guides for immunoglobulin A nephropathy in Japan, third version (the fraction of the total number of glomeruli displaying active or chronic lesions are <25%: grade I, 25%-49.9%: grade II, 50%-74.9%: grade III, and ≧75%: grade IV).

### 2.4 Outcomes

The primary outcome was the occurrence of renal events, defined as the first occurrence of ≧30% decrease in eGFR from baseline.^[15,16]^ Secondary outcomes were the incidences of clinical remission, relapse and adverse events. Urinary remission was defined as cessation of hematuria (<5 RBC/HPF) or proteinuria (<0.3 g/gCr) 3 or more consecutive times or over 6 months,^[17]^ respectively. The cessation of both hematuria and proteinuria was defined as clinical remission. We also assessed the incidences of relapse among patients with urinary remission following TSP. Relapse was defined as reappearance of urinary abnormalities^[18]^ in 3 consecutive analyses.

### 2.5 Statistical analysis

To summarize baseline characteristics, we used mean ± standard deviation for continuous variables, and numbers and percentages (%) for categorical variables. We compared normally distributed (or non-parametric), categorical variables using unpaired Student *t* test and Pearson χ^2^ test, respectively. The cumulative incidences of the decrease in eGFR, remission, and recurrence in the 2 groups were analyzed using the Kaplan-Meier method and compared using a Log-rank test. We used Cox proportional hazards regression models to evaluate the impacts of baseline factors on renal outcome (TSP1 vs TSP3, age over mean vs under mean, male vs female, eGFR <60 vs ≧60, proteinuria ≧0.5 g/gCr vs <0.5 g/gCr, hematuria score ≧6 vs <6, H-grade ≧2 vs 1, alb over mean vs under mean and RAS-i vs no-RAS-i). All independent variables used in Cox regression analyses were categorical variables. The results of analyses were expressed as hazard ratios (HR) with 95% confidence intervals (CI). All analyses were performed using JMP Pro 14 (SAS Institute Inc, Tokyo, Japan), and GraphPad Prism 7 software (GraphPad, La Jolla, CA). Power calculations were based on a comparison of the 2 treatment groups using the Southwest Oncology Group statistical tools (Seattle, WA). Differences with *P* values of <.05 (bilateral) were considered significant.

## 3 Results

### 3.1 Patient characteristics

**Figure 1:**
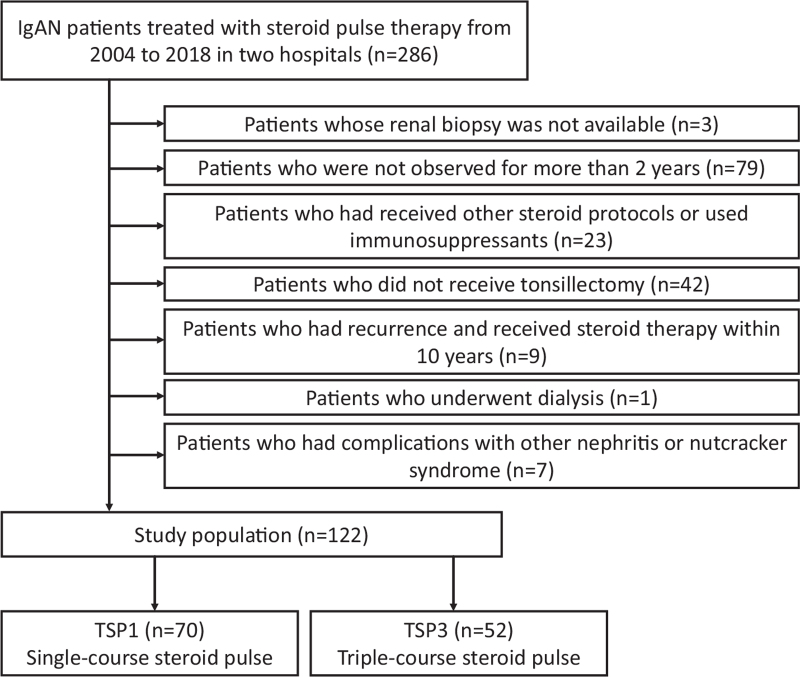
Flow chart of patient selection. IgAN = immunoglobulin A nephropathy, TSP1 = tonsillectomy combined with single-course corticosteroid pulse, TSP3 = tonsillectomy combined with triple-course corticosteroid pulse.

We enrolled 286 IgAN patients who underwent corticosteroid pulse therapy from 2004 to 2018. We excluded 3 patients whose renal biopsies were unavailable, 79 who were not followed-up for over 2 years, 23 who had received other corticosteroid protocols or immunosuppressants, 42 who did not undergo tonsillectomy, 9 who had a recurrence of the disease and received steroid therapy within 10 years, 1 on hemodialysis, and 7 complicated with other renal diseases. Among the remaining 122 patients, 70 underwent single-course steroid pulse therapy (TSP1), whereas 52 underwent triple-course steroid pulse therapy (TSP3) combined with tonsillectomy (Fig. 1).

The baseline characteristics of the 2 groups are shown in Table 1. There were no significant differences in gender, age, body mass index, systolic and diastolic blood pressure, IgA, C3, IgA/C3, hemoglobin A1c between the 2 groups. Mean levels of Alb were significantly higher in TSP1 (4.1 g/dL) than in TSP3 (3.7 g/dL). Mean levels of proteinuria (TSP1 vs TSP3: 0.75 vs 1.07 g/gCr), sCr (0.93 vs 0.86 mg/dL), eGFR (78.5 vs 76.2 mL/min/1.73 m^2^), the proportion of hematuria score over 6 (i.e. ≧30-49/HPF) (40.0% vs 44.2%), distribution of CKD stage and clinical grade (defined by Japanese Society of Nephrology), and the interval from renal biopsy had no significant difference between the 2 groups. The rate using RAS-i in TSP1 (30.0%) was higher than in TSP3 (13.5%). Histological grade was not significantly different between the 2 groups (Table 2).

**Table 1:** Comparison of baseline characteristics between Group TSP1 and TSP3.

	TSP1 (n = 70)	TSP3 (n = 52)	*P* value
Male	31 (44.3)	16 (30.8)	.14
Age (yrs)	36.1 ± 12.2	36.0 ± 11.2	.97
BMI (kg/m^2^)	22.0 ± 3.5	21.4 ± 3.4	.34
Interval from renal biopsy (yrs)	1.6 ± 4.1	0.6 ± 1.4	.06
Observation period (yrs)	4.8 ± 1.8	5.4 ± 1.8	.06
Systolic BP (mm Hg)	118.3 ± 15.7	116.9 ± 15.0	.64
Diastolic BP (mm Hg)	74.0 ± 11.4	74.5 ± 13.3	.85
Hematuria score ≧6	28 (40.0)	23 (44.2)	.71
Proteinuria (g/gCr)	0.75 ± 0.83	1.07 ± 1.18	.08
sCr (mg/dL)	0.93 ± 0.48	0.86 ± 0.33	.38
eGFR (mL/min/1.73 m^2^)	78.5 ± 31.2	76.2 ± 24.6	.67
CKD G1+2	46 (65.7)	38 (73.1)	
CKD G3a	16 (22.9)	9 (17.3)	
CKD G3b	5 (7.1)	4 (7.7)	
CKD G4	2 (2.9)	1 (1.9)	
CKD G5	1 (1.4)	0 (0)	
Clinical grade			.38
Grade 1	35 (50.0)	20 (38.5)	
Grade 2	20 (28.6)	16 (30.8)	
Grade 3	15 (21.4)	16 (30.8)	
Alb (g/dL)	4.1 ± 0.4	3.7 ± 0.4	.0001
IgA (mg/dL)	332.5 ± 157.9	299.4 ± 119.9	.22
C3 (mg/dL)	100.2 ± 20.4	100.7 ± 19.0	.90
IgA/C3 ratio	3.4 ± 1.5	3.0 ± 1.3	.21
HbA1c (%)	5.3 ± 0.6	5.4 ± 0.3	.41
RAS-I	21 (30.0)	7 (13.5)	.049

Data are mean ± SD or number (%). Alb = albumin, BMI = body mass index, BP = blood pressure, C3 = complement 3, CKD = chronic kidney disease, eGFR = estimated glomerular filtration rate, HbA1c = hemoglobin A1c, IgA = immunoglobulin A, RAS-i = renin-angiotensin system inhibitor, sCr = serum creatinine, TSP = tonsillectomy combined with corticosteroid pulse.

**Table 2:** Histological grade.

	TSP1	TSP3	*P* value
Oxford classification	n = 27	n = 31	
Mesangial score (M1)	5 (18.5)	5 (16.1)	1.00
Segmental glomerulosclerosis (S1)	16 (59.3)	19 (61.3)	1.00
Endocapillary hypercellularity (E1)	6 (22.2)	10 (32.3)	.56
Tubular atrophy/interstitial fibrosis (T)			.68
T0	25 (92.6)	27 (87.1)	
T1	2 (7.4)	4 (12.9)	
T2	0 (0)	0 (0)	
Histological grade (Japanese Society of Nephrology)	n = 63	n = 45	.30
I	32 (50.8)	15 (33.3)	
II	20 (31.8)	17 (37.8)	
III	9 (14.3)	11 (24.4)	
IV	2 (3.2)	2 (4.4)	

Data are number (%). TSP = tonsillectomy combined with corticosteroid pulse.

### 3.2 Outcomes of renal survival and clinical remission

**Figure 2:**
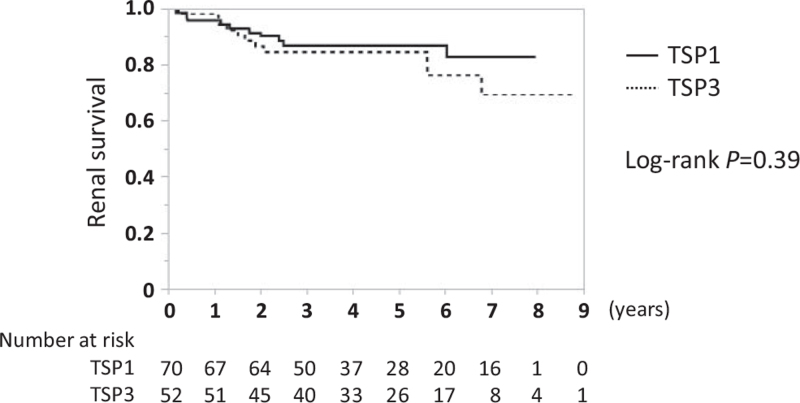
Comparison of renal survival between TSP1 and TSP3. The primary outcome for renal survival (≧30% decrease in eGFR from baseline) in the 2 groups was analyzed using the Kaplan-Meier method and log rank test. eGFR = estimated glomerular filtration rate, TSP1 = tonsillectomy combined with single-course corticosteroid pulse, TSP3 = tonsillectomy combined with triple-course corticosteroid pulse.

**Figure 3:**
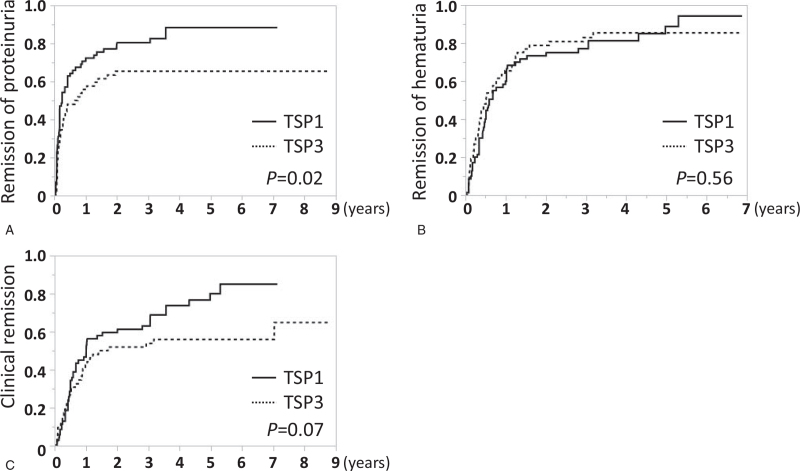
Cumulative incidence of remission of hematuria, proteinuria, and both (clinical remission) in TSP1 and TSP3. (A) Cumulative incidence of remission of proteinuria (less than 0.3 g/gCr in 3 consecutive analyses or over 6 mo) in the 2 groups. (B) Cumulative incidence of remission of hematuria (less than 5 RBC/HPF in 3 consecutive analyses or over 6 mo) in the 2 groups. (C) Cumulative incidence of clinical remission (remission of both hematuria and proteinuria) in the 2 groups. Comparisons were made using the Kaplan-Meier method and log rank test. Statistically significant differences between the groups are indicated with *P* < .05. HPF = high power field, TSP1 = tonsillectomy combined with single-course corticosteroid pulse, TSP3 = tonsillectomy combined with triple-course corticosteroid pulse.

**Figure 4:**
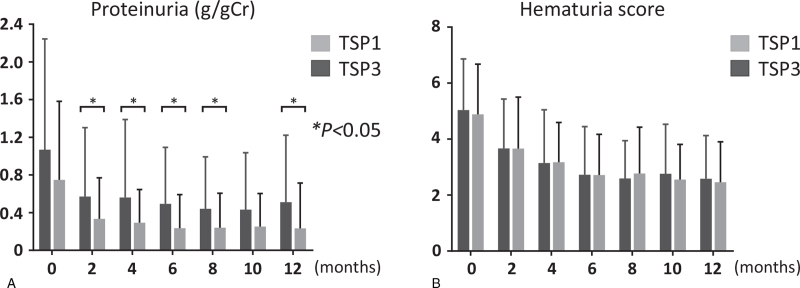
Serial changes of proteinuria and hematuria in TSP1 and TSP3. (A) The levels of proteinuria (g/gCr) in the 2 groups were described from the baseline to 12 mo. (B) The hematuria scores in the 2 groups were described from the baseline to 12 mo. Data represent the mean ± standard deviation. Comparisons were made using an unpaired Student *t* test for each month. Statistically significant differences between the groups are indicated with asterisks, ^∗^*P* < .05. TSP1 = tonsillectomy combined with single-course corticosteroid pulse, TSP3 = tonsillectomy combined with triple-course corticosteroid pulse.

Eight-years renal survival (defined as ≧30% decrease in eGFR from baseline) was 82.5% and 69.2%, in TSP1 and TSP3, respectively, with a statistical power of over 90% (2-sided, α = 0.05); the difference was not significant (Fig. 2). The cumulative incidences of remission of hematuria, proteinuria, and both (clinical remission) in TSP1 and TSP3 groups were 94.4% and 85.4%, 88.4% and 65.4%, and 85.0% and 64.8%, respectively. While there were no significant differences between the 2 groups in the cumulative incidences of remission of hematuria and clinical remission, those of proteinuria showed higher rates in TSP1 than TSP3 (Fig. 3). Among patients who achieved urinary remission, the mean intervals from baseline to remission of hematuria were 0.91 years in TSP1 and 0.67 years in TSP3, and those of proteinuria were 0.52 years in TSP1 and 0.44 years in TSP3. Regarding the clinical course, the levels of hematuria in the 2 groups showed no significant differences within 12 months. The levels of proteinuria were significantly lower in TSP1 than in TSP3 at 2, 4, 6, 8, and 12 months (Fig. 4).

**Figure 5:**
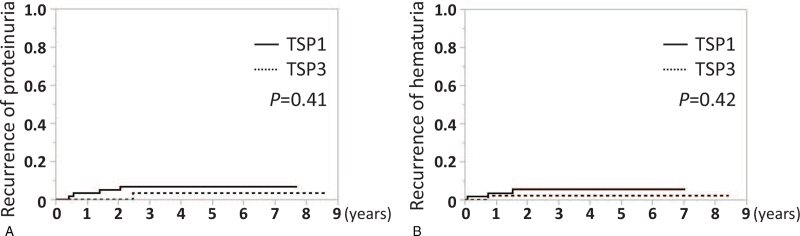
Cumulative incidence of relapse of hematuria and proteinuria in TSP1 and TSP3. (A) Cumulative incidence of relapse of proteinuria (more than 0.3 g/gCr in 3 consecutive analyses, followed by additional treatment to re-induce remission) in the 2 groups. (B) Cumulative incidence of relapse of hematuria (more than 5 RBC/HPF in 3 consecutive analyses, followed by additional treatment to re-induce remission) in the 2 groups. Comparisons were made using the Kaplan-Meier method and log rank test. Statistically significant differences between the groups are indicated with *P* < .05. HPF = high power field, TSP1 = tonsillectomy combined with single-course corticosteroid pulse, TSP3 = tonsillectomy combined with triple-course corticosteroid pulse.

We analyzed the relapse rate among patients who achieved urinary remission after undergoing TSP1 or TSP3. The number of patients who achieved remission of hematuria and proteinuria was 56 and 58 in TSP1, and 44 and 34 in TSP3, respectively. The cumulative incidences of relapse of hematuria and proteinuria were 5.6% and 7.1% in TSP1, and 2.3% and 3.3% in TSP3, respectively. Overall, there were no significant differences between the 2 groups (Fig. 5). Among patients who experienced urinary relapse, the mean duration from remission to relapse of hematuria and proteinuria was 0.79 years and 1.11 years in TSP1, and 0.75 years and 2.45 years in TSP3, respectively. All patients with relapse underwent additional treatment.

During the TSP treatment, no serious adverse event that required hospitalization occurred in the 2 groups.

### 3.3 Associated baseline factors contributing to renal outcome

We used Cox proportional hazards regression analysis to identify the factors associated with renal outcome (≧30% decrease in eGFR from baseline) among all patients who underwent TSP treatment (Table 3). Univariate Cox regression analysis revealed that age, gender, number of courses of corticosteroid pulse therapy (TSP1 vs TSP3: HR 0.69, 95% CI 0.29-1.64, *P* = .39), severity of histological grade, levels of eGFR, proteinuria, hematuria, and RAS-i use (RAS-i vs no-RAS-i: HR 1.80, 95% CI 0.68-4.34, *P* = .22) did not influence renal outcome.

**Table 3:** Univariate Cox regression analysis of factors associated with renal outcome (≧30% decrease in eGFR from baseline).

Variable (at baseline)	Hazard ratio	95% CI	*P* value
TSP1	0.69	0.29-1.64	.39
Age over mean (>36)	0.49	0.19-1.19	.12
Male	0.81	0.32-1.95	.66
eGFR <60	0.95	0.36-2.31	.92
Proteinuria ≧0.5g/gCr	1.07	0.45-2.62	.88
Hematuria score ≧6	0.73	0.28-1.76	.49
H-grade ≧2	0.72	0.19-2.29	.58
Alb over mean (>3.9)	0.95	0.40-2.27	.90
RAS-I	1.80	0.68-4.34	.22

Alb = albumin, CI = confidence intervals, eGFR = estimated glomerular filtration rate, RAS-i = renin-angiotensin system inhibitor, TSP = tonsillectomy combined with corticosteroid pulse.

## 4 Discussion

In this study, we compared the efficacies of single- and triple-course corticosteroid pulse therapy combined with tonsillectomy in patients with IgAN. Our findings demonstrated that a single-course protocol (TSP1) achieved favorable outcomes, including preservation of renal function and induction of urinary remission, not significantly different from the triple-course protocol (TSP3) with adequate statistical power. In addition, TSP1 and TSP3 showed comparable efficacy to prevent relapse after remission. These results indicate that the TSP1 protocol may improve the prognosis of patients with IgAN as the conventional TSP protocol (TSP3).

The pathogenesis of IgAN is characterized by the mesangial deposition of IgA1 immune complexes,^[19]^ which is associated with a disordered mucosal-bone marrow immunity and initiates glomerular injury leading to glomerulosclerosis and tubulo-interstitial fibrosis.^[2,20]^ Considering these pathologies, tonsillectomy might be useful for regulating the upstream of the disease via reducing aberrant IgA1, whereas corticosteroid therapy has the potential to ameliorate renal injury, the downstream of IgAN pathogenesis, by suppressing glomerular inflammation and fibrosis. Although either single treatment of corticosteroid or tonsillectomy has been reported to have insufficient or contradictory results,^[21–24]^ the combined tonsillectomy and corticosteroid pulse therapy introduced by Hotta et al^[9]^ showed favorable outcomes. A prospective controlled study showed that the combined tonsillectomy and corticosteroid pulse therapy was superior to steroid pulse monotherapy in inducing clinical remission and preserving renal function via the maintenance of urinary remission.^[24]^ Therefore, the combined therapy has since been widely used in Japan. Recently, a large retrospective cohort study demonstrated favorable long-term outcomes of tonsillectomy independent of the use of corticosteroid and RAS-i^[8]^ and the indication of TSP treatment for patients with IgAN has been accepted.

However, the dosage of corticosteroids remains controversial, thus an optimal regimen of corticosteroid administration in TSP protocol is needed for avoiding excessive adverse effects.^[11,12,25]^ Some reports focused on the number of courses of corticosteroid pulse therapy combined with tonsillectomy. Kaneko et al^[26]^ reported that single-course corticosteroid pulse therapy combined with mizoribine was effective for preserving renal function compared to triple-course corticosteroid pulse therapy. The study indicated that the addition of mizoribine might reduce the required number of courses of corticosteroid pulse therapy. Watanabe et al^[27]^ showed that TSP3 had an effect to rapidly induce remission of hematuria compared to TSP1, although there was no significant difference in the clinical remission rates between the 2 groups, which was consistent with our study. Their study was conducted over a short term and did not assess renal outcome. Takada et al^[28]^ reported that tonsillectomy with triple-course corticosteroid pulse therapy had a relation to better renal prognosis compared to twice or fewer courses of corticosteroid pulse therapy. However, the analysis did not directly assess the differences in efficacies between TSP1 and TSP3. Furthermore, the timing of corticosteroid pulses was not uniformed. Their study settings were therefore different from ours.

In the present study, we directly evaluated the clinical remission and renal prognosis of TSP1 and TSP3 therapy in which the corticosteroid pulse was conducted within a limited period. The primary outcome for renal survival and the incidence of clinical remission showed no significant difference in the 2 groups with adequate statistical power (i.e. >90%, 2-sided, α = 0.05), implying that TSP1 protocol might be sufficient for effective regulation of IgAN in certain cases. Of note, we investigated the relapse rate following TSP treatment. The relapse rate after corticosteroid monotherapy was roughly 20% to 50%,^[29]^ while the rate after tonsillectomy alone was around 40% at 2 years after in remission, according to the previous reports. In our study, TSP3, and notably TSP1, showed lower relapse rates compared to these reports. This preventive effect can be explained by the mechanisms of tonsillectomy as reducing the chance of developing chronic tonsillitis, decreasing the production of pathogenic IgA,^[7]^ and increasing CD4^+^ CD25^+^ Treg cells.^[30]^ Furthermore, corticosteroid pulse therapy can reduce IgA1 production via inducing apoptosis of memory T lymphocytes.^[31]^ Based on these therapeutic mechanisms, the combination of tonsillectomy and even only a single-course corticosteroid pulse is efficient to reset the vicious cycle of the mucosa-bone marrow axis, resulting in the prevention of relapse. This corticosteroid-minimized therapy can contribute to shorter hospitalization periods. Moreover, the smaller number of corticosteroid pulses might prevent side effects including osteoporosis,^[32]^ hyperglycemia,^[33]^ and psychosis.^[34]^ Although randomized controlled clinical trials are needed to confirm the effectiveness of the therapy, our report indicates the possibility of TSP1 treatment as an effective and safe treatment strategy for IgAN.

There are some limitations in this study. First, this is a retrospective cohort study with a limited sample size, which may have led to bias. Importantly, the selection of the number of corticosteroid pulse therapy was determined by physicians. In fact, TSP3 group at baseline had hypo-albuminemia compared to TSP1, which may underestimate the superiority of TSP3. However, there was no significant difference in the levels of proteinuria and renal function at baseline between the 2 groups. Second, the efficacy of TSP1 in cases with severe IgAN, who rapidly progress to renal dysfunction with high disease activity, is uncertain because the majority of enrolled IgAN patients in this study presented with mild activity and chronic course. Third, we recorded only serious adverse events related to TSP. Additional prospective cohort studies are required to further validate the efficacies and disadvantages of single-course corticosteroid pulse administration combined with tonsillectomy.

## 5 Conclusions

Our study demonstrated that single-course corticosteroid pulse administration combined with tonsillectomy achieved favorable outcomes, including preservation of renal function and induction of urinary remission, and prevented relapse, as triple-course protocol.

## Acknowledgments

We thank all the staff members at Hokkaido University Hospital and NTT Medical Center Sapporo who were taking care of patients. We thank Richard Robins, PhD, from Edanz Group (https://en-author-services.edanzgroup.com/) for editing a draft of this manuscript.

## Author contributions

KW-K, DN, and NK contributed to the conception and design of the work. KW-K and DN wrote and summarized the manuscript. KW-K, DN, JY, NM, NK, TN, RY, TM, and SN managed and followed up the patients’ care. SI, TT, and YF made the pathological diagnosis. KW-K, DN, and NN made statistical analyses. KW-K, DN, JY, TN, SI, TT, SN, and TA revised the manuscript critically. All authors have read and approved the manuscript.

**Conceptualization:** Kanako Watanabe-Kusunoki, Daigo Nakazawa.

**Formal analysis:** Kanako Watanabe-Kusunoki, Naoki Nishimoto.

**Investigation:** Kanako Watanabe-Kusunoki, Junya Yamamoto, Sari Iwasaki, Takahiro Tsuji, Yuichiro Fukasawa, Saori Nishio.

**Resources:** Tasuku Nakagaki, Rie Yamamoto, Tomochika Maoka.

**Validation:** Junya Yamamoto.

**Writing – original draft:** Kanako Watanabe-Kusunoki.

**Writing – review & editing:** Daigo Nakazawa, Naoko Matsuoka, Nobuharu Kaneshima, Tatsuya Atsumi.
